# Clinical Usefulness of the Serological Gastric Biopsy for the Diagnosis of Chronic Autoimmune Gastritis

**DOI:** 10.1155/2012/520970

**Published:** 2012-11-29

**Authors:** Antonio Antico, Marilina Tampoia, Danilo Villalta, Elio Tonutti, Renato Tozzoli, Nicola Bizzaro

**Affiliations:** ^1^Laboratorio Analisi Chimico-Cliniche, Ospedale Civile, 35013 Cittadella, Italy; ^2^Laboratorio di Patologia Clinica, Azienda Ospedaliero-Universitaria, Policlinico di Bari, 70124 Bari, Italy; ^3^Allergologia e Immunologia Clinica, Azienda Ospedaliera S. Maria degli Angeli, 33170 Pordenone, Italy; ^4^Allergologia e Immunopatologia, Azienda Ospedaliero-Universitaria S. Maria della Misericordia, 33100 Udine, Italy; ^5^Laboratorio di Patologia Clinica, Azienda Ospedaliera S. Maria degli Angeli, 33170 Pordenone, Italy; ^6^Laboratorio di Patologia Clinica, Ospedale San Antonio, 33028 Tolmezzo, Italy

## Abstract

*Aim*. To assess the predictive value for chronic autoimmune gastritis (AIG) of the combined assay of anti-parietal-cell antibodies (PCA), anti-intrinsic-factor antibodies (IFA), anti-*Helicobacter pylori* (Hp) antibodies, and measurement of blood gastrin. *Methods*. We studied 181 consecutive patients with anemia, due to iron deficiency resistant to oral replacement therapy or to vitamin B12 deficiency. *Results*. 83 patients (45.8%) tested positive for PCA and underwent gastroscopy with multiple gastric biopsies. On the basis of the histological diagnosis, PCA-positive patients were divided into 4 groups: (1) 30 patients with chronic atrophic gastritis; they had high concentrations of PCA and gastrin and no detectable IFA; (2) 14 subjects with metaplastic gastric atrophy; they had high PCA, IFA, and gastrin; (3) 18 patients with nonspecific lymphocytic inflammation with increased PCA, normal gastrin levels, and absence of IFA; (4) 21 patients with multifocal atrophic gastritis with “borderline” PCA, normal gastrin, absence of IFA and presence of anti-Hp in 100% of the cases. *Conclusions*. The assay of four serological markers proved particularly effective in the diagnostic classification of gastritis and highly correlated with the histological profile. As such, this laboratory diagnostic profile may be considered an authentic “serological biopsy.”

## 1. Introduction

Chronic autoimmune gastritis (AIG), also known as type A chronic atrophic gastritis, is an organ-specific disease that causes malabsorption of essential elements and pernicious or microcytic anemia, and it is a predisposing factor to carcinoid tumour and gastric adenocarcinoma. It is generally asymptomatic up to an advanced stage of atrophy and/or dysplasia of the mucosa [1, 2]. Histologically, it is characterised by a chronic inflammatory disorder of the gastric body and the fundus, sustained by a cell-mediated aggression by CD4^+^CD25^−^ Th1 lymphocyte effectors. The main target of immunological injury is the H^+^/K^+^-adenosine-triphosphate enzyme (ATPase), a protein of the membrane that coats the secretory canaliculi of the parietal cells and is responsible for the secretion of the hydrogen ions in exchange for the potassium ions (proton pump) [3, 4]. Induced by a triggering factor not yet entirely identified, the CD4^+^CD25^−^  T-cells, together with macrophages and B lymphocytes, infiltrate the submucosa, the lamina propria, and the gastric glands causing the loss of parietal, principal, and ghrelin-producing cells or P/D1 cells [5, 6]. The damage of the body and fundus mucosa results in [3, 7]

- hypo/achlorhydria, as a result of destruction of the parietal cells;
- hypergastrinemia, as a result of alteration of the negative feedback mechanism modulated by gastric acidity and governed by somatostatin via paracrine mechanism;
- malabsorption, which causes iron-deficiency anemia resistant to oral treatment or vitamin B12-deficiency anemia;
- presence of circulating autoimmune antibodies directed against the proton pump and the intrinsic factor, a consequence of the effector cytokine action of the T lymphocytes on humoral immunity;
- reduction of serum levels of pepsinogen and ghrelin, the principal and P/D1 cells being destroyed as bystanders of the parietal cells.

The presence of immunological markers and/or the change in the biochemical markers may be indicative of autoimmune gastritis, even in a clinically asymptomatic subject. Moreover, since these markers appear long before the clinical symptoms in the natural history of the disease, their assay ought to make it possible to identify AIG in an earlier phase [8, 9]. 

The purpose of our study was to assess the predictive value (VP) for AIG of the combined testing for anti-parietal-cell antibodies (PCA), anti-intrinsic-factor antibodies (IFA), anti-*Helicobacter pylori* (Hp) antibodies and gastrin, in patients suffering from iron-deficiency or vitamin B12 deficiency anemia, and to analyse the diagnostic efficacy of this laboratory profile in the selection of the subjects candidate to endoscopy.

## 2. Patients and Methods

Over the span of 14 months, 181 consecutive patients were recruited (19 males and 162 females; age: 25–81 years), referred by their GPs with diagnosis of microcytic iron-deficiency anemia resistant to oral treatment (39.8%) or vitamin B12 deficiency macrocytic anemia (60.2%), clinical conditions that are correlated to AIG [3]. None of the patients had bleeding of the gastroenteric tract nor, in the case of the female sex, menometrorrhagia, or liver disease, chronic inflammatory gut disease, or other diseases causing malabsorption. In all patients, blood cell count and the microscopic analysis of the peripheral blood smear showed moderate anisopoikilocytosis of the erythrocytes and, in 67% of the cases of macrocytic anemia, hypersegmentation of the neutrophils. 

The assay of serum iron and vitamin B12 was carried out using enzymatic and immunological methods, respectively, on automated analysers with detection by modular structure enhanced chemiluminescence (Modular P and Modular E 170, Roche Diagnostics, Basle, Switzerland). 

In the 181 selected patients the following markers were assayed:

- anti-parietal-cell antibodies (PCA), using quantitative immunoenzymatic method (ELISA) (Aesku.Diagnostics, Wendelsheim, Germany (cutoff value: 30 U/mL));
- anti-intrinsic-factor antibodies (IFA), using quantitative ELISA method (Aesku.Diagnostics—cutoff value: 20 U/mL);
- gastrin, using automated quantitative immun-chemiluminescent method (Immulite 2000, Siemens HealthCare Diagnostics, Flanders, NY, USA—reference interval: 35–115 pg/mL);
- anti-*Helicobacter pylori* antibodies (anti-Hp) using quantitative ELISA method (Orgentec Diagnostika, Mainz, Germany—cutoff value: 6 U/mL).

The cut-off values adopted were those suggested by the manufacturers.

The study protocol scheduled that the recruited subjects that presented positivity for PCA and/or IFA should, after informed consent, be subjected to esophagogastroduodenoscopy (EGDS), irrespective of the levels of gastrinemia. The site and number of the biopsy samples were decided in accordance with recent recommendations [1, 10, 11]: one biopsy from the greater curvature of the fundus, two biopsies from the greater curvature of the body, and two biopsies from the lesser curvature of the body, that is, from the sites involved in the chronic inflammation process typical of AIG. The statistical analysis was carried out using Student's *t*-test for nonpaired data and the statistics software SPSS 13.0 (Chicago, IL, USA).

## 3. Results

Eighty-three out of 181 patients (45.8%) tested positive for PCA, 14 of them (16.8%) had also positive IFA. Among the 69 patients who were positive for PCA only, 36 (52.2%) had microcytic, and 33 (47.8%) had macrocytic anemia. In the 14 patients with positivity for both PCA and IFA, microcytic anemia was present in 4 cases (28.6%) and macrocytic anemia in 10 cases (71.4%). Forty-four patients out of 83 (53%) had raised gastrin, whereas gastrin was normal in all the seronegative subjects. Anti-Hp antibodies were present in 33 (39.7%) patients. All 83 PCA-positive subjects and, as control, 11 PCA-negative subjects with gastrin within the normal range, referred by the GP to the gastroenterologist for recurrent dyspeptic disturbances, underwent EGDS with multiple gastric biopsies. On the basis of the histological diagnosis it was possible to divide the patients into four groups (Table 1): Group 1: 30 (36%) patients (mean age: 53 ± 20) with a histological profile of chronic atrophic gastritis of body and fundus; Group 2: 14 (17%) subjects (mean age: 70 ± 16) with metaplasic gastric atrophy of body and fundus, histological lesion more severe and extensive than that observed in Group 1; Group 3: 18 (22%) patients (mean age: 46 ± 12) had nonspecific lymphocytic gastritis; Group 4: 21 patients (25%) (mean age: 71 ± 12) had multifocal atrophic gastritis.

Serological analysis showed that Group 1 patients had high concentrations of PCA (mean 65 U/mL), high gastrin (mean 1048 pg/mL), absence of IFA, and presence of anti-Hp antibodies in 26% of the cases; Group 2 patients had an average concentration of PCA of 59 U/mL, gastrin of 1523 pg/mL, positivity for IFA, and absence of anti-Hp; Group 3 patients had an average concentration of PCA of 52 U/mL, normal gastrin, absence of IFA, and presence of anti-Hp in 21.1% of the cases; Group 4 patients had an average concentration of PCA of 18 U/mL (SD ± 8), normal IFA and gastrin, and all were anti-Hp positive (Table 1). 

The 11 sero-negative subjects showed normal levels of gastrin and a histological profile of superficial gastritis.

## 4. Discussion

In the natural history of many autoimmune diseases, specific autoantibodies may be present in the preclinical or subclinical phase of the disease, when the functions of the target organ are conserved or offset by homeostatic mechanisms [12, 13]. In AIG too, it is possible to hypothesise that the appearance of specific antibodies may precede by many years the onset of clinical symptoms, the increase of serum gastrin, the decrease of A and C pepsinogens and ghrelin, and the deficiency of iron and vitamin B12. However, observations on the predictive role of PCA and IFA in the natural history of autoimmune gastritis are relatively rare and discordant [14–16]. Nonetheless, a recent prospective study carried out on patients with autoimmune thyroid diseases demonstrated that, after 5 years, 24% of the subjects who were PCA and IFA positive at the time of enrolment developed histologically diagnosed AIG [17]. The same study also showed that the concentration of PCA increases progressively up to a peak, followed by a decrease as a result of ongoing destruction of the gastric mucosa. Our results show that PCA and IFA assays can be predictive of AIG and are useful in the selection of patients to be referred for diagnostic procedures. We consecutively enrolled 181 patients with microcytic iron-deficiency anemia resistant to oral treatment or macrocytic anemia due to vitamin B12 deficiency (known as frequently related to AIG in otherwise asymptomatic individuals), referring over the span of 14 months to our Laboratory Medicine Service. On patients' sera we measured PCA, IFA, and gastrin, as markers of gastric mucosa damage. We also included anti-Hp antibodies since, in addition to being considered as predictive of mucosa damage, they also correlate with the presence of PCA in patients with AIG, possibly due to molecular mimicry between gastric ATPase and bacterial urease [18–21].

The histological diagnosis after EGDS allowed to classify the patients in 4 subgroups.

In Group 1, histology showed chronic atrophic gastritis of the body and fundus. The lamina propria and the glandular tissue were infiltrated by mononuclear cells (T and B lymphocytes and macrophages). The T-cell-mediated epithelial lesion, mainly at the level of parietal cells, results in reduction of gastric acid secretion and consequently impairment of the control mechanism inhibiting gastrin secretion by G-cells of the antrum. In fact, these patients have PCA positivity and raised gastrin; this serological profile, in subjects with iron- or vitamin B12-deficiency anemia, is highly predictive of autoimmune lesion of the stomach mucosa. 

In Group 2, histology showed metaplastic atrophy of body and fundus, more severe than that observed in Group 1, with extensive lymphocyte, monocyte, and neutrophil mucosa infiltration. The lesions were extended to over 50% of glands in the total area of the biopsy material: areas of pseudopyloric metaplasia were also present. As shown in Table 1, the quantitative assay of PCA and gastrin was unable to discriminate AIG from gastric atrophy. However, PCA values were slightly lower in atrophy, presumably because of a more extensive parietal cell destruction [17, 22]. Instead, the presence of IFA in this group of patients correlated with a more serious histological lesion and therefore has likely a greater predictive value for gastric atrophy. None of these subjects was positive for anti-Hp; the destruction of gastric epithelium, which is necessary for the survival of Hp, prevents the bacteria from colonising and proliferating in the stomach [23]. Negativity for anti-Hp associated with positivity for PCA, IFA and raised gastrin in patients with anemia increases the positive predictive value for the diagnosis of gastric atrophy. 

Group 3 patients showed lymphocytic gastric inflammation, nonspecific histological lesion characterised by infiltration of the gastric mucosa by mononuclear cells without glandular damage. They showed presence of PCA, normal gastrin, absence of IFA, and in some cases presence of anti-Hp. Group 4 patients had atrophic gastritis of the antrum and body, with focal intestinal metaplasia of the glands, caused by Hp, and showed absence of PCA and IFA, normal gastrin, and presence of anti-Hp.

Therefore, based on our data, normal serum gastrin and the absence of IFA have a fair negative predictive value for autoimmune lesion of the mucosa of the stomach, as demonstrated by correlating the biopsy profile with the laboratory results in the 39 subjects of Groups 3 and 4; they tested positive for PCA only (notably, at very low values in those with gastritis due to Hp), but showed no lesions of gastric epithelium. On the contrary, the rise of gastrin and the presence of IFA, in association with PCA, are highly predictive for AIG and atrophy, as apparent from the data obtained in the first two groups of patients.

Thus, although these results should be confirmed in several larger studies, the determination of the 4 laboratory markers (PCA, IFA, anti-Hp, and gastrin) could be regarded as a “serological biopsy” useful in selecting patients candidate to EGDS (Table 2).

Testing for PCA and IFA using ELISA method features elevated analytical and diagnostic accuracy, provides continuous quantitative results, and improves standardisation of the analytic procedure, compared to discrete quantitative or qualitative tests (such as indirect immunofluorescence and immunoblotting). Moreover, it makes it possible to monitor the disease, with special reference to mucosal atrophy development [22]. The anti-Hp assay increases the diagnostic value of the “serological biopsy.” It should be noted that patients with nonspecific gastritis were younger than those with AIG, but with comparable PCA and anti-Hp positivity; this suggests the need to monitor the patients showing such histological profile by serology, for example, once a year, since AIG may occur in some of them, especially if they are genetically predisposed, such as first-degree relatives of patients with AIG or subjects with genetic variations at the *ABO* gene locus [24, 25].

In conclusion, autoimmune gastritis is a frequently asymptomatic disease that can cause atrophy of the gastric mucosa and in approximately 10% of cases evolve into a carcinoid tumour or adenocarcinoma [1, 26]. In the absence of clinical symptoms, only the reasoned use of laboratory tests makes it possible to clearly classify the patient with suspected AIG to be subjected to endoscopic examination. The presence of risk factors and/or of therapy-resistant B12 or iron-deficient anemia, in the absence of aetiological agents that can be directly correlated, is clinical criteria that suggest to test for PCA, IFA, and gastrin as immunological and biochemical markers of AIG. This profile can be considered as an authentic “serological biopsy” [27–29] considering that the hypergastrinemia identifies the damage to the mucosa of the body and fundus of the stomach, and the presence of the antibodies proves the autoimmune origin of the aggression of the mucosa itself. Indeed, the data obtained in our study demonstrate that the positivity for PCA and elevated gastrin correlate with damage to the mucosa of the body and of the fundus typical of AIG. The IFA positivity indicates the presence of a more serious histological profile (atrophy). Significant values of PCA, in the absence of IFA and an increase in gastrin, are associated with nonspecific lymphocytic inflammation rather than with autoimmune damage to the gastric mucosa. Finally, the elevated predictive value and the clinical specificity of the “serological biopsy” are confirmed by the evidence that none of the patients with normal gastrin and absence of IFA revealed lesions of the gastric mucosa of an autoimmune type.

## Figures and Tables

**Table 1:** Analytic data of the four serological markers in relation to histological diagnosis.

Histological diagnosis	Number of patients	Age (years) (mean ± SD)	PCA (U/mL) (mean ± SD) (cutoff 30)	IFA (U/mL) (mean ± SD) (cutoff 20)	Gastrin (pg/mL) (mean ± SD) (cutoff 115)	Anti-Hp positivity (%)
Group 1: autoimmune gastritis	30	53.5 ± 20.0	65 ± 29	12 ± 2	1048 ± 956	26.0
Group 2: gastric atrophy	14	70.4 ± 16.5*	59 ± 23	67 ± 8*	1523 ± 713*	0*
Group 3: non-specific lymphocytic gastritis	18	45.7 ± 12.0	52 ± 17	5 ± 4	38 ± 8*	21.1
Group 4: multifocal atrophic gastritis	21	70.6 ± 11.9*	18 ± 8*	6 ± 6	56 ± 11*	100*

PCA: anti-parietal-cell antibodies; IFA: anti-intrinsic-factor antibodies; anti-Hp: anti-*Helicobacter  pylori* antibodies; *statistical significance (*P* < 0.05).

**Table 2:** Biochemical-antibody results in relation to the histological diagnosis of gastritis in the studied population.

Histological diagnosis	PCA	IFA	Gastrin	Anti-Hp
Autoimmune gastritis	+	−	+	±
Gastric atrophy	+	+	+	−
Non-specific lymphocytic gastritis	+	−	−	±
Multifocal atrophic astritis	−	−	−	+

## References

[1] Dixon MF, Genta RM, Yardley JH, Correa P (1996). Classification and grading of gastritis. The updated Sydney System. International Workshop on the Histopathology of Gastritis, Houston 1994. *American Journal of Surgical Pathology*.

[2] De Block CEM, De Leeuw IH, Van Gaal LF (2008). Autoimmune gastritis in type 1 diabetes: a clinically oriented review. *Journal of Clinical Endocrinology and Metabolism*.

[3] Toh BH, Van Driel IR, Gleeson PA (1997). Mechanisms of disease: pernicious anemia. *The New England Journal of Medicine*.

[4] Arnold R (2007). Diagnosis and differential diagnosis of hypergastrinemia. *Wiener Klinische Wochenschrift*.

[5] Asano M, Toda M, Sakaguchi N, Sakaguchi S (1996). Autoimmune disease as a consequence of developmental abnormality of a T cell subpopulation. *Journal of Experimental Medicine*.

[6] Taguchi O, Takahashi T (1996). Administration of anti-interleukin-2 receptor *α* antibody in vivo induces localized autoimmune disease. *European Journal of Immunology*.

[7] Martinelli TM, Van Driel IR, Alderuccio F, Gleeson PA, Toh BH (1996). Analysis of mononuclear cell infiltrate and cytokine production in murine autoimmune gastritis. *Gastroenterology*.

[8] Correa P (2004). Is gastric cancer preventable?. *Gut*.

[9] Jevremovic D, Torbenson M, Murray JA, Burgart LJ, Abraham SC (2006). Atrophic autoimmune pangastritis: a distinctive form of antral and fundic gastritis associated with systemic autoimmune disease. *American Journal of Surgical Pathology*.

[10] Tucci A, Bisceglia M, Rugge M (2007). Clinical usefulness of gastric-juice analysis in 2007: the stone that the builders rejected has become the cornerstone. *Gastrointestinal Endoscopy*.

[11] Sjoblom SM, Sipponen P, Jarvinen H (1993). Gastroscopic follow up of pernicious anaemia patients. *Gut*.

[12] Scofield RH (2004). Autoantibodies as predictors of disease. *The Lancet*.

[13] Bizzaro N, Tozzoli R, Shoenfeld Y (2007). Are we at a stage to predict autoimmune rheumatic diseases?. *Arthritis and Rheumatism*.

[14] Leslie D, Lipsky P, Notkins AL (2001). Autoantibodies as predictors of disease. *Journal of Clinical Investigation*.

[15] Tozzoli R (2008). The diagnostic role of autoantibodies in the prediction of organ-specific autoimmune diseases. *Clinical Chemistry and Laboratory Medicine*.

[16] Villalta D, Tozzoli R, Tonutti E, Bizzaro N (2007). The laboratory approach to the diagnosis of autoimmune diseases: is it time to change?. *Autoimmunity Reviews*.

[17] Tozzoli R, Kodermaz G, Perosa AR (2010). Autoantibodies to parietal cells as predictors of atrophic body gastritis: a five-year prospective study in patients with autoimmune thyroid diseases. *Autoimmunity Reviews*.

[18] Hershko C, Ronson A, Souroujon M, Maschler I, Heyd J, Patz J (2006). Variable hematologic presentation of autoimmune gastritis: age-related progression from iron deficiency to cobalamin depletion. *Blood*.

[19] Amedei A, Bergman MP, Appelmelk BJ (2003). Molecular mimicry between helicobacter pylori antigens and H^+^ ,K^+^-adenosine triphosphatase in human gastric autoimmunity. *Journal of Experimental Medicine*.

[20] D’Elios MM, Appelmelk BJ, Amedei A, Bergman MP, Del Prete G (2004). Gastric autoimmunity: the role of Helicobacter pylori and molecular mimicry. *Trends in Molecular Medicine*.

[21] Presotto F, Sabini B, Cecchetto A (2003). Helicobacter pylori infection and gastric autoimmune diseases: is there a link?. *Helicobacter*.

[22] van Driel IR, Baxter AG, Laurie KL (2002). Immunopathogenesis, loss of T cell tolerance and genetics of autoimmune gastritis. *Autoimmunity Reviews*.

[23] Oksanen A, Sipponen P, Karttunen R (2000). Atrophic gastritis and Helicobacter pylori infection in outpatients referred for gastroscopy. *Gut*.

[24] Varis K, Ihamaki T, Harkonen M (1979). Gastric morphology, function, and immunology in first-degree relatives of probands with pernicious anemia and controls. *Scandinavian Journal of Gastroenterology*.

[25] Nakao M, Matsuo K, Ito H (2011). ABO genotype and the risk of gastric cancer, atrophic gastritis, and Helicobacter pylori infection. *Cancer Epidemiology Biomarkers and Prevention*.

[26] Antico A (2008). La gastrite autoimmune. *RIMeL/IJLaM*.

[27] Korstanje A, den Hartog G, Biemond I, Lamers CBHW (2002). The serological gastric biopsy: a non-endoscopical diagnostic approach in management of the dyspeptic patient: significance for primary care based on a survey of the literature. *Scandinavian Journal of Gastroenterology, Supplement*.

[28] Storskrubb T, Aro P, Ronkainen J (2008). Serum biomarkers provide an accurate method for diagnosis of atrophic gastritis in a general population: the Kalixanda study. *Scandinavian Journal of Gastroenterology*.

[29] Burkitt MD, Varro A, Pritchard DM (2009). Importance of gastrin in the pathogenesis and treament of gastric tumors. *World Journal of Gastroenterology*.

